# Fitness in CLL

**DOI:** 10.3390/cancers18020342

**Published:** 2026-01-21

**Authors:** Claudia Baratè, Ilaria Scortechini, Sara Ciofini, Paola Picardi, Ilaria Angeletti, Federica Loscocco, Alessandro Sanna, Alessandro Isidori, Elisa Grazioli, Paolo Sportoletti

**Affiliations:** 1 Department of Hematology, Niguarda Cancer Center, ASST Grande Ospedale Metropolitano Niguarda, 20161 Milano, Italy; claudia.barate@gmail.com; 2 Clinic of Hematology, Azienda Ospedaliero Universitaria delle Marche, 60126 Ancona, Italy; ilaria.scortechini@ospedaliriuniti.marche.it; 3 Department of Cell Therapies, Hematology Unit, Senese Hospital and University, 53100 Siena, Italy; sara.ciofini@ao-siena.toscana.it; 4 Department of Hematology and Cellular Therapy, Mazzoni Hospital, 63100 Ascoli Piceno, Italy; paola.picardi@sanita.marche.it; 5 Department of Onco-Hematology, Terni Hospital, 05100 Terni, Italy; ilariaangeletti@yahoo.it; 6 Hematology and Stem Cell Transplant Center, AST Pesaro and Urbino, 61121 Pesaro, Italy; federica.loscocco@gmail.com (F.L.); alessandro.isidori@sanita.marche.it (A.I.); 7 Hematology Unit, AOU Careggi, 50134 Firenze, Italy; sannaa@aou-careggi.toscana.it; 8 Unit of Physical Exercise and Sport Sciences, Department of Movement, Human and Health Sciences, University of Rome Foro Italico, 00135 Roma, Italy; elisa.grazioli@uniroma4.it; 9 Department of Medicine and Surgery, Institute of Hematology and Center for Hemato-Oncology Research (CREO), University of Perugia, Santa Maria della Misericordia Hospital, 06132 Perugia, Italy

**Keywords:** chronic lymphocytic leukemia, fitness, biological age, nutrition, physical activity, comorbidities

## Abstract

The increased complexity of CLL management, driven by the advent of targeted therapies, makes it necessary to redefine the concept of fitness, which until a few years ago was limited to the patient’s ability to undergo chemoimmunotherapy. In the current therapeutic landscape, fitness assessment must also consider biological age, comorbidities, and frailty, which have become increasingly relevant due to evolving treatment options and a more nuanced understanding of the elderly CLL population. In this context, the general condition of the patient should be preserved with greater attention to physical activity and nutrition. This narrative review analyzed in depth all these aspects with the aim of providing insights into fitness assessments and their actualization.

## Simple Summary

Treating CLL has become more complex because of new targeted therapies. As a result, the idea of “fitness” needs to be redefined. In the past, fitness mainly meant whether a patient could tolerate chemotherapy and immunotherapy. Today, fitness also considers biological age, comorbidities, polypharmacy and overall frailty. These factors are especially important as treatments continue to change and as we better understand older patients with CLL. Therefore, more attention should be given to maintaining a patient’s overall health, including physical activity and nutrition. This review examines these topics in detail and aims to clarify how fitness should be evaluated and updated in current clinical practice.

## 1. Introduction

Chronic lymphocytic leukemia (CLL) predominantly affects older adults, and for decades, chronological age has been the principal criterion used to define patient fitness and guide treatment decisions [1,2,3]. However, modern approaches to evaluating whether a patient is “fit” or “unfit” are beginning to move beyond rigid chronological age assessments. Chronological age, in contrast to biological age—which better reflects overall health status and the heterogeneity of aging—often fails to capture the multidimensional factors that influence treatment outcomes, tolerance, and quality of life, including functional status, comorbidity burden, cognitive function, nutritional status, polypharmacy, and social support.

Indeed, aging-related conditions, infections, and cardiovascular events significantly contribute to mortality in patients with CLL, often independently of disease status, and translate into a substantial reduction in life expectancy compared with the general population [4]. To better stratify patients, clinicians have adopted various scoring systems such as the Cumulative Illness Rating Scale (CIRS) [5], the Charlson Comorbidity Index (CCI), and the Eastern Cooperative Oncology Group (ECOG) performance status—a clinician-assessed scale based on functional capacity and daily activity level—[6] and creatinine clearance (CC) [7,8]. These widely used tools often fail to provide a sufficiently granular view of patient fitness and are sometimes inadequate in guiding optimal treatment selection.

In recent years, the therapeutic landscape of CLL has shifted dramatically, with a growing preference for chemotherapy-free regimens that include continuous or time-limited therapies with targeted agents. In this new context, traditional fitness scores appear increasingly outdated and insufficient to identify the most appropriate treatment strategy, as illustrated in several real-world studies [9].

Fitness assessment should go beyond age and comorbidities to incorporate additional patient-centered factors, including polypharmacy, physical and cognitive function, nutritional status, performance of daily activities, and the availability of social support.

This evolution parallels the emerging needs in CLL, where diverse treatment modalities require individualized selection based on broader patient factors.

This narrative review aims to outline the current state of the art in fitness assessment in CLL and to explore the limitations of existing tools. We advocate for a multidimensional, dynamic model of fitness that integrates geriatric and frailty assessments, exercise capacity, and nutritional status, emphasizing the role of interdisciplinary professionals such as kinesiologists and nutritionists in optimizing patient care in the era of precision medicine.

## 2. Fitness in Acute Myeloid Leukemia: Transferable Lessons for CLL?

The experience gained in acute myeloid leukemia (AML) provides a rational basis for redefining the concept of fitness in other hematologic malignancies, including CLL. In AML, the assessment of patient fitness plays a pivotal role in guiding treatment decisions by identifying patients who can safely receive intensive therapy, tailoring treatment strategies, and minimizing treatment-related toxicity.

In AML, several clinical scores have been developed and employed to determine eligibility for intensive chemotherapy, tailor therapeutic strategies, and reduce treatment-related mortality [9]. Among AML scores, the Ferrara criteria are the most widely used and very good at predicting treatment mortality [10]. As a purely clinical score, the Ferrara criteria have the potential for bias due to clinician discretion and do not consider biology; therefore, they cannot be used to predict overall survival.

Importantly, the therapeutic landscape in AML has evolved significantly with the introduction of novel, less intensive regimens. In this context, the notion of fitness is shifting from a binary classification of fit versus unfit for intensive chemotherapy to a more nuanced concept of fitness for a specific treatment.

In addition, age is not a barrier to the beneficial effects of treatment in AML [11], and selected patients in their 80s and 90s may achieve responses and improve survival when treated with intensive therapy [12,13]. Now, there is a need for patient-centered care that emphasizes the importance of incorporating quality of life, self-reported physical and social functioning, and social support into the assessment toolkit, as well as the early integration of palliative care.

These observations suggest that while tools like the Ferrara score provide a useful framework, they are not sufficient to capture the full complexity of patient fitness. The CLL field can draw from these experiences but must go further, developing multidimensional approaches tailored to the unique biology and treatment dynamics of the disease.

## 3. The Role of Age in Defining Fitness

Worldwide, life expectancy increased dramatically from 34 years in 1913 to 74.8 years in 2025, and it is expected to rise further in the coming decades [14]. Notably, the global share of individuals aged ≥80 is projected to rise to almost 5% by mid-century [14]. Statistics highlight the growing incidence of CLL with age, particularly in the elderly population, representing roughly 20% of the general CLL population [15,16]. Shortly, the number of elderly patients with CLL will continue to rise, prompting the medical community to redefine the concept of age in fitness criteria. For many years, chronological age has been a cornerstone of fitness assessment in oncology, including CLL. The WHO traditionally defined ‘elderly’ based on chronological age: 60–74 years as young elderly, 75–89 years as old elderly, and 90 years and older as very old elderly. However, more recent approaches emphasize functional health status, focusing on physical, cognitive, and psychological well-being rather than age alone, and age thresholds, such as 65 years, are increasingly being challenged [3]. As a result, chronological age provides only a partial view of a patient’s true fitness for therapy [3]. While advanced age may serve as an immediate indicator of susceptibility to certain side effects, it fails to account for crucial variables such as organ function, comorbidities, physical reserve, and psychological resilience. Therefore, chronological age does not capture how “biologically young” an individual is in terms of physical and cognitive capacity. This phenomenon has become increasingly evident in CLL, where many elderly patients tolerate innovative treatments with good outcomes, despite their age. This shift enables the personalization of treatment for patients who might otherwise be excluded from effective therapies based solely on their age.

Biological age, which may differ significantly from chronological age, offers a more dynamic and personalized perspective on a patient’s overall health. It encompasses physiological parameters and biomarkers that reflect organ and tissue health, as well as resilience and stress response capacity [17,18]. Approaches incorporating biological age markers, such as frailty indices and functional tests (e.g., walking speed and handgrip strength), provide a more accurate view of treatment tolerance and adverse event risk than chronological age alone.

Frailty encompasses physical weakness, weight loss, and low levels of physical activity, and it has been linked to worse outcomes in CLL. Studies show that frail patients, even those with similar chronological ages, tend to experience more severe side effects from treatment and have a poorer prognosis [19].

The presence of comorbidities such as cardiovascular disease, diabetes, and renal impairment can severely affect how well patients tolerate treatments, even in the era of targeted therapies.

In addition to physical health, cognitive function, closely related to physical fitness, is another important factor to consider. Cognitive impairment was associated with worse treatment adherence and outcomes in older CLL patients, even when there were no significant comorbidities [20,21].

Together, these studies make it clear that a patient’s biological age is a more accurate predictor of treatment outcomes in CLL than chronological age alone. Incorporating biological age measurements into clinical protocols can enhance therapeutic personalization, enabling potentially more effective treatments even for chronologically older patients.

## 4. Limits of Comorbidities Score Systems: How to Cross Them to Redefine Fitness

Different clinical tools are available to evaluate fitness in patients with cancer, aimed at differentiating biological ages from chronological age, looking for frailty and vulnerability to antineoplastic treatments, and identifying elderly patients free of comorbidities, functionally independent, who could benefit from intensive treatments without an increase in toxicity (Table 1). To date, no single clinical tool has emerged as the most suitable, as available instruments incompletely capture the multidimensional nature of fitness, particularly in the era of targeted therapies. Several clinical tools are currently employed to evaluate fitness in CLL, each characterized by specific merits and demerits. CIRS [5,22,23], which systematically assesses 1 integrity across 14 organ systems, remains the historical gold standard for clinical trials due to its validated ability to predict survival; however, its primary demerit is its complex and time-consuming execution, which often limits its utility in routine practice. To overcome these practical barriers, the CLL-Comorbidity Index (CLL-CI) [4,24,25] was developed by focusing only on three high-impact organ systems (vascular, endocrine, and upper gastrointestinal). Its main merit lies in its simplicity and speed, and it has proven to be an independent predictor of event-free survival (EFS) even in patients treated with targeted agents like ibrutinib. In contrast, while the ECOG PS [6,8,9,26,27] is universally adopted for its reproducibility and ease of use, its significant demerit is its lack of granularity, as it fails to capture multidimensional factors such as cognitive function, nutritional status, and social support. The Comprehensive Geriatric Assessment (CGA) [28,29] offers a holistic evaluation of functional and psychosocial domains, successfully identifying “invisible” frailties and predicting drug discontinuation; nevertheless, its demerit involves its complex clinical implementation, and its specific role in the era of novel targeted therapies requires further prospective validation. Finally, the CCI [30,31,32] is effective for predicting non-CLL-related mortality, yet it fails to correlate with disease-specific survival or treatment-related toxicities.

CIRS and PS are the most widely used scores for CLL, both as part of the inclusion criteria for clinical trials and as predicting factors of survival in patients treated with chemoimmunotherapy or target agents, regardless of biological factors. Fitness assessment has historically played a key role in guiding the choice of chemoimmunotherapy for CLL [16], with the CIRS score being the most used tool for this purpose [5]. A CIRS score of ≥6 or a CrCl of <70 mL/min is used to classify patients as “unfit” or less suitable for intensive chemotherapy, such as fludarabine, cyclophosphamide, and rituximab (FCR) [15,32,35].

Consequently, patients with high CIRS scores or impaired renal function were historically considered unsuitable for intensive fludarabine-based chemoimmunotherapy because of an increased risk of toxicity and treatment-related complications. For this reason, they were preferentially treated with less intensive regimens, including chlorambucil-based therapies, which were associated with a more favorable tolerability profile in this patient population [16,36].

The introduction of targeted therapies, such as Bruton Tyrosine Kinase (BTK) inhibitors and BCL2 inhibitors, has significantly transformed the treatment landscape. Compared to chemoimmunotherapy, these agents have lower myelotoxicity and are more tolerable in older patients or those with comorbidities [6,7,30,37,38]. However, they are associated with specific toxicities, such as cardiovascular or bleeding events, which can be exacerbated by pre-existing comorbidities, potentially leading to dose reductions or treatment discontinuation.

In the context of targeted therapies, defining fitness is challenging. Currently, a CIRS score > 6 and CrCl < 70 mL/min are still used as surrogate markers for fitness.

Pivotal trials included patients deemed unfit for FCR; however, most participants had relatively few comorbidities and preserved renal function. Notably, the fixed-duration trials specifically enrolled patients with significant comorbidities.

In all five trials, fitness was not recognized as a negative prognostic factor. However, clinical trial populations are inherently selective, with patients with ECOG scores >2 being systematically excluded (Table 2).

Targeted therapy consistently performed better than chemoimmunotherapy, regardless of ECOG-PS or CIRS scores [6,7,30,37,38]. In a pooled analysis of CLL13 and CLL14 studies, patients randomized to venetoclax + obinutuzumab were stratified as fit or unfit (CIRS > 6 and/or CrCL < 70 mL/min); the 3-year-PFS rates were 86.4% in unfit vs. 87.5% in fit patients (HR 1.12, 95%-CI 0.70–1.81, *p* = 0.63), thus indicating that the treatment was feasible regardless patient’s fitness. The incidence of toxicities was comparable between groups, although injection-related reactions and fatigue were more common in fit patients. Reduced venetoclax dose intensity had no impact on PFS, suggesting that dose modifications may have a limited impact on long-term clinical outcomes [43].

Prospective data evaluating the impact of baseline fitness on clinical outcomes, treatment tolerability, and survival in patients receiving targeted therapies remain limited. However, retrospective analyses provide valuable insights into the prognostic role of ECOG-PS and CIRS in patients receiving ibrutinib and venetoclax.

A prospective cohort study including 3306 patients with R/R CLL treated with ibrutinib showed that refractory disease, the number of prior lines of therapy, age, reduced renal function, and ECOG-PS were independently associated with shorter time to treatment discontinuation (TTD) and overall survival (OS) in a multivariate analysis [44]. In 95 patients enrolled in the Swedish compassionate program of ibrutinib, CIRS ≥ 6 was associated with a shorter progression-free survival (PFS) and OS [45]. Similar outcomes were reported in a retrospective series of 145 patients, where the CIRS score was associated with decreased event-free survival (EFS) and OS in patients treated in frontline or salvage therapy. CIRS > 6 impacted survival also in patients younger than 65 years, while in patients ≥ 65 years, when CIRS scores were <6, a single severe organ impairment (score of 3 or 4) was significantly associated with decreased OS [46].

Consistently, an Italian retrospective cohort of 712 patients treated with ibrutinib in the real world confirmed the independent role of CIRS > 6 in reducing treatment tolerance, PFS, and EFS, but not OS. Notably, according to age, comorbidities predicted a significantly shorter PFS and EFS in elderly patients [9].

Another retrospective study described the impact of age, fitness, disease characteristics, and concomitant medications on outcomes in CLL patients receiving venetoclax and identified ECOG-PS as the only factor correlating with PFS, EFS, and OS [47].

A comorbidity score alone, such as the CIRS or CLL-CI, may not be sufficient to accurately define the patient’s fitness, as these tools do not fully capture functional status, frailty, or other relevant clinical dimensions; it may, however, integrate a more comprehensive geriatric assessment. A multidimensional CGA conducted on 75 out of 97 patients enrolled in a clinical trial with fludarabine treatment showed that cognitive impairment significantly correlated with shorter EFS, and functional and cognitive impairments were associated with reduced OS [48]. Similarly, in a real-world cohort of 108 patients with CLL, a simplified CGA, including age, CIRS, and physical function, was used to stratify patients into frail, pre-frail, and fit categories and successfully predicted OS and time-to-first-treatment (TTFT), independently of Rai stage. In this cohort, as recognized by the authors, most patients received outdated therapies, such as chlorambucil-based treatments (56.8%), with only six (10.3%) receiving ibrutinib [49].

The Alliance (A041202) clinical trial applied an optional CGA component and Cancer and Aging Research Group Chemotherapy Toxicity Tool (CARG-TT) [50], in 369 out of 524 participants. Specific CGA domains, rather than the full assessment, successfully predicted drug discontinuation, PFS, and OS. Social support influenced both PFS and OS after adjusting for baseline characteristics and treatment arms, while nutritional status impacted only PFS. Protective factors against drug discontinuation included low depression and anxiety levels, high social activity scores, and minimal weight loss in the preceding six months. Notably, the CARG-TT did not predict treatment-related toxicity in any arm of the study, and frail patients in the ibrutinib arms demonstrated improvements across geriatric domains over time [51].

In the CLL-Frail trial, the FRAIL scale score ≥ 3 was used with age ≥ 80 years to enroll patients to be treated with acalabrutinib monotherapy. The FRAIL scale score is a 5-item self-assessment questionnaire for patients correlating with Fried’s frailty phenotype [52]. In this study, estimated 12-month PFS and OS rates were 87.5% and 91.9%, respectively, and 53% of patients who received at least three cycles of therapy self-assessed an improvement in FRAIL scale scores, with 21% of patients considered frail at month 6 compared to 47% at screening [53].

In a prospective Mayo Clinic study of 1143 CLL patients, comorbidities at diagnosis were systematically assessed to determine their impact on survival and cause of death. Patients with higher CCI scores or a greater number of major comorbidities had shorter non-CLL-specific survival, whereas CLL-specific survival was not affected on multivariate analysis. These results indicate that deaths in CLL patients were primarily driven by leukemia and its complications, regardless of baseline comorbidity burden. Nevertheless, a history of stroke, cardiac disease, or higher overall CCI scores was associated with an increased likelihood of death from non-CLL-related causes [54].

Even if comorbidity indices demonstrated their correlation with EFS and OS, the most common causes of death in CLL patients are disease progression (46%), infections (8%), and second cancers (19%) [54,55]; in non-CLL deaths, cardiovascular, high CCI score and number of comorbidities are critical for survival [54].

In the Danish CLL registry, renal and psychiatric disease had the highest weight in all-cause mortality, and more than half of the comorbidity patients died from CLL-related causes, without receiving any antineoplastic treatments [56].

Clinical evidence suggests an association between CLL and chronic illness, with a more aggressive CLL profile in presence of comorbidities [57,58,59]. Although the mechanisms underlying this increased aggressiveness are not clear, some of the working hypotheses include direct organ infiltration, the activity of the microenvironment, the role of chronic inflammation, immune dysfunction, the carcinogenesis linked to dysmetabolic diseases, and alterations in the microbiome [54,59,60,61,62].

Based on the correlation between comorbidity and outcomes in CLL patients but not in CLL-related mortality, comorbidities indexes could be useful in: (i) predicting the most likely cause of death after diagnosis and life-expectancy due to comorbidities; (ii) helping management of coexisting conditions to reduce CLL-unrelated mortality and specific-organ-toxicity; and (iii) receiving better caregiver counseling, improving the number of comorbid patients receiving treatment and their compliance to treatment, without excluding from treatment comorbid patients who could benefit from antineoplastic treatment (e.g., high-risk CLL). The optimum would be a fitness score able to predict the correlation between comorbidity and treatment toxicity, both to make accessible therapy, even in clinical trials, to patients who could truly tolerate the treatment, and to guide the best treatment schedule (e.g., dose reduction).

In summary, while CIRS and CrCl remain valuable tools, there is a growing need for individualized fitness assessments to optimize treatment selection. Future studies should integrate CGA, frailty indices, and patient-reported outcomes to better capture the heterogeneity of the CLL population. CGA, encompassing functional, cognitive, and psychosocial domains, has proven useful in predicting treatment tolerance and survival in older adults with CLL. However, its role in the targeted therapy era warrants further investigation.

## 5. Do the Guidelines Actualize the Concept of Fitness?

Regarding first-line therapy, ESMO guidelines recommend a time-limited therapy, especially the combination of venetoclax and obinutuzumab for patients with favorable biological characteristics (IgHV mutation and TP53 wild type), regardless of age or fitness [3]. Conversely, 12 cycles of ibrutinib–venetoclax (preceded by ibrutinib for three months) are recommended only for fit and/or younger patients, regardless of IGHV mutational status, and should be considered with caution for older patients with cardiac comorbidities. This position comes from the results of the GLOW trial [38] that included an older/unfit population and showed a survival benefit over chemoimmunotherapy, burdened with few cardiac or sudden early deaths. The CAPTIVATE trial, which nevertheless enrolled young patients aged less than 70 years, did not report this kind of fatal event [41]. Continuous therapy with BTK inhibitors, especially acalabrutinib or zanubrutinib, is recommended for patients with TP53 deletion or mutation, regardless of fitness or age. BTK inhibitors are related to adverse events of special interest, which include cardiovascular events, hypertension, and atrial fibrillation; zanubrutinib [39] and acalabrutinib [31] show a better safety profile and are the preferred option.

Infusion-related reactions during the first cycles and tumor lysis syndrome are potentially serious adverse events related to obinutuzumab + venetoclax, which is avoided in patients with severe kidney disease and less preferred in patients with concomitant nephrotoxic medications [8,40].

Therefore, in front-line CLL, risk stratification has been recently rethought: in the era of targeted therapy, fitness, and age have less relevance than in the past, as the evaluation of comorbidities, concomitant therapies, and logistical aspects can better guide therapeutic choice.

## 6. Polypharmacy and Drug Interactions: Is It an Outdated Concept?

The presence of comorbidities often requires the administration of concomitant therapies that may interfere with CLL treatments. The primary route of metabolism and elimination of most BTK inhibitors and venetoclax is through the cytochrome P450 3A (CYP3A)-mediated pathway, which plays a prominent role in the metabolism of numerous other drugs and natural substances [63,64,65,66]. The dose of BTK inhibitors must be decreased when they are administered with strong inhibitors of CYP3A4 and closely monitored for side effects. It is also important to avoid, when possible, concomitant use of strong inducers of CYP3A4 because of the lack of drug efficacy in disease control (Table 3) [67,68,69,70].

A novel oral formulation of acalabrutinib has overcome the interaction with proton pump inhibitors, which determined variable absorption.

Pharmacological interaction between BTK inhibitors and cardiovascular drugs deserves a separate discussion, due to the large prevalence of cardiovascular disease in the elderly population. Some widely used drugs, like verapamil, amiodarone, and diltiazem, are strong or moderate inhibitors of CYP3A4 and should be avoided during treatment with BTK inhibitors, especially ibrutinib. Ibrutinib could raise plasma levels of dabigatran and digoxin in patients with atrial fibrillation and may inhibit P-glycoprotein and the human breast cancer resistance protein transport when administered at recommended doses [69].

**Figure 1:**
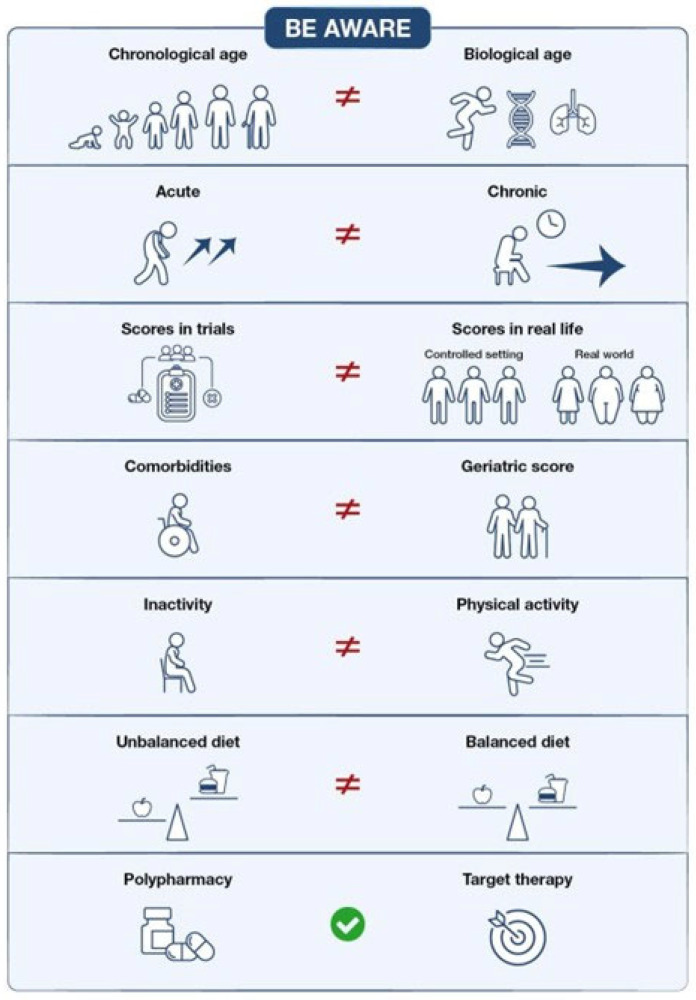
Factors that should be considered during clinical assessment of patients with CLL. Clinicians should be aware that a broader and multifactorial concept of fitness considers biological age, comorbidities, geriatric scores, ability to perform physical activity, nutritional status, and the need for polypharmacy to provide a comprehensive assessment of patients with CLL.

Therefore, the treatment of CLL with new target therapies has become simpler but determines more pharmacological interactions that should be continuously monitored. As not only drugs but also foods (grapefruit juice and Seville oranges, St John’s wort) could have a dangerous interaction with CLL treatment, patient education on dangerous eating habits can ensure a proper and safe administration of CLL therapies (Figure 1).

## 7. Role of Exercise in Redefining Fitness and the Figure of the Kinesiologist

Before discussing in depth the role of exercise and physical activity in defining the fitness of patients with CLL, it is necessary to clearly define the two terms, which are not synonymous.

According to the WHO, “physical activity is any movement determined by the musculoskeletal system that results in energy expenditure greater than in resting conditions.” Included in this definition are simple movements such as walking, bicycling, dancing, playing, gardening, and housework, which are part of “spontaneous motor activity” [71].

The term “exercise” refers to planned, structured, repetitive, and oriented physical activity to maintain one or more of the components of fitness (endurance, strength, power, joint flexibility/mobility) and/or improve specific parameters (balance, proprioception, dexterity, etc.) [72].

Another fundamental concept is the definition of sedentary activities, which are those characterized by very low energy expenditure, exemplified by sitting or reclining (i.e., watching television, driving a car, reading, working at a desk, etc.). These types of behaviors negatively affect fitness parameters by quickening the process of decline, especially in elderly patients [73].

According to WHO guidelines for individuals over the age of 65, moderate-intensity aerobic physical activity for at least 150–300 min or intense aerobic physical activity for 75–150 min should be performed. To this should be added muscle-strengthening exercises, two or more times a week. In addition, to maintain good fitness and prevent falls, multi-component physical activity, which is a combination of aerobic activity, muscle strengthening, and balance training, should be performed at least three days a week. Suggested levels of physical activity for sick patients are like those for non-sick peers, within the limits of what is compatible with individual functional capacity [74].

Physical activity alone is not enough unless sedentariness is also controlled. Sedentariness and physical inactivity are not two equivalent concepts. A person can be physically active and achieve the recommended amount of physical activity, but simultaneously be sedentary [75]: for example, people who do 20 min of running in the morning but then spend the rest of the day in a car or sitting at a desk. This is why the scientific community recommends frequent active breaks during sedentary periods, at least 2–3 min every 30 min [76].

To improve fitness parameters, it is necessary to follow a well-structured exercise protocol adapted to individual functional capacity. The Preventive and Adapted Motor Activities Kinesiologist (AMPA Kinesiologist) intervenes precisely in this area and, following the exercise prescription made by the competent physician, performs a functional assessment, with specific tests to evaluate endurance, strength, power, joint flexibility/mobility, and balance. With the results of these tests, the kinesiologist can structure a feasible and effective workout for the patient [77].

Patients with CLL during the treatment-naive period may have low overall fitness and specific physical dysfunction, both of which predict poor survival after treatment initiation [78]. For this reason, the best approach to increasing physical fitness and immune function simultaneously may be participating in a structured and adapted exercise program supervised by a kinesiologist [77].

Increased levels of physical activity and, consequently, fitness parameters improved physical efficiency and quality of life, as well as reduced therapy-related side effects in patients with lymphoma [79]. In another study, a 36-week combined training course could improve parameters such as strength and balance, as well as patients’ quality of life during treatments [80]. However, these studies include only 11.5% of patients with CLL and 46% of patients with B-cell non-Hodgkin’s lymphoma. This makes it necessary to further investigate with specific studies, the role of exercise on fitness parameters, survival, and prognosis of patients with CLL.

**Figure 2:**
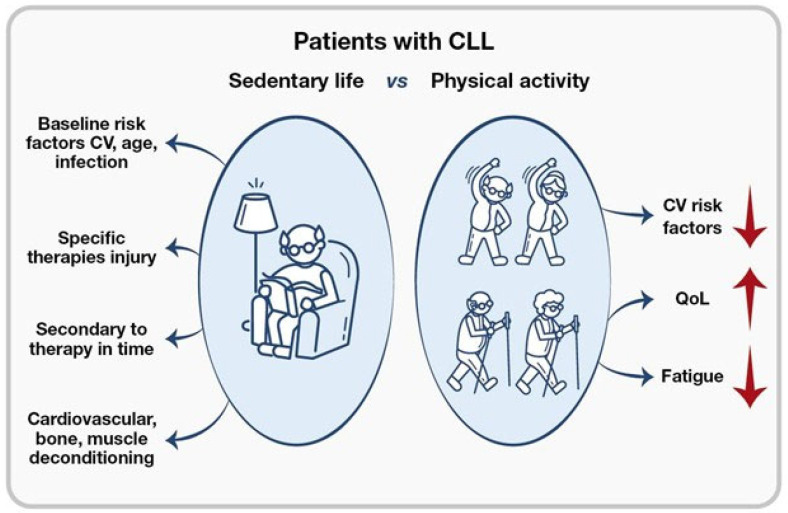
Personal approach to physical activity. Physical activity provides several benefits beyond the decrease in cardiovascular risk, including the amelioration of quality of life and a decreased perception of fatigue. It is essential for the multidisciplinary team that cares for patients with CLL to include a kinesiologist.

A personal approach to physical activity should be defined in CLL to improve patient motivation and control disease-specific adverse events. During “therapy holidays”, physical activity should be encouraged to reduce cardiovascular factors and improve performance and outcomes in case of a need for a new therapy. Therapy efficacy depends on the quality of life and the maintenance of a physical and mental state during and after treatment. In all disease settings, a personalized rehabilitation program can contribute to restoring physical and psychosocial functioning. For elderly patients, it should be useful to contrast the reduction in activity, which affects everyday activities and prevents falls and cardiovascular events (Figure 2).

## 8. Nutrition and the Role of the Nutritionist in Redefining Fitness

Nutritional imbalances affect health and may result in long-term effects if not controlled early. Malnutrition is defined as a state of functional, structural, and developmental change in the body, resulting from a discrepancy between nutrient requirements, intake, and utilization. It is common in the elderly for inadequate or protein-calorie undernutrition, together with mineral and vitamin deficiencies. Indeed, during life, the body undergoes a change in its composition with a decrease in muscle mass, an increase in fat tissue, and reduced hydration and bone mineralization. With age, the thirst signal decreases and the sense of satiety increases, leading to a reduction in food intake. A category at greater risk of malnutrition is the frail elderly, i.e., those of advanced or very advanced age, suffering from multiple chronic diseases, clinically unstable, often disabled, with socio-economic problems such as loneliness and poverty, whose nutritional status and ability to provide adequate nutrition are often compromised [81,82].

Nutritional deficiencies are a common problem in patients with cancer and are linked to poorer response to treatment, more toxicities, more clinical symptoms and complications, reduced quality of life, and poorer overall outcomes [83].

Mild to moderate malnutrition can be treated with a balanced diet, to which supplements may be added, especially if there are difficulties with solid foods and multivitamin supplements. To ensure a correct diet for the elderly, in addition to the nutrients mentioned above, it is necessary to provide proteins with a high biological value that are useful for maintaining lean mass, such as meat, fish, and eggs; the consumption of legumes provides essential micronutrients such as iron and calcium. It is also important to limit the consumption of simple sugar and promote the proper amounts of complex carbohydrates, such as those present in pasta, rice, barley, and fresh bread, to preserve the carbohydrate intake [84,85].

Protein-energy malnutrition, along with other predisposing factors, often leads to sarcopenia, a progressive and generalized loss of skeletal muscle mass and strength or function that is commonly associated with aging. Sarcopenia significantly increases the risk of numerous adverse health outcomes, including falls, morbidity, loss of independence, disability, and even mortality. However, nutritional interventions, both alone and in combination with physical activity programs, can help prevent and, in many cases, even reverse sarcopenia [86,87].

Overnutrition, in which calorie intake exceeds the body’s needs, is another form of malnutrition, leading to overweight (BMI of 25.1–29.9) and obesity (BMI over 30), and an increased risk of cardiovascular disease and diabetes [86]. In recent years, as confirmed by the WHO, the increasing number of cases of obesity associated with diabetes has led clinicians to define a “new” pathological condition called “diabesity” [88].

Addressing diabesity requires multifaceted strategies, with diet playing a pivotal role [87]. Underlying this condition are several risk factors that are part of the more common metabolic syndrome, linked to a sedentary lifestyle and a diet characterized by excessive consumption of saturated fats, simple sugars, and salt. Treatment of metabolic syndrome is based on a multidisciplinary approach that includes the use of conventional medications (especially for those at high cardiovascular risk), nutraceuticals (in combination or not with conventional medications), and lifestyle changes (diet, physical activity, reduced alcohol consumption, and cigarette smoking) [89,90].

Since the risk of cardiovascular adverse events, including atrial fibrillation, hypertension, and heart failure, is not negligible during the treatment with BTK inhibitors [91], a pre-treatment assessment should include a comprehensive medical history, a targeted cardiovascular assessment to identify the main risk factors present, and early nutritional screening to evaluate the presence of obesity or metabolic syndrome should be recommended before starting any specific therapy.

**Figure 3:**
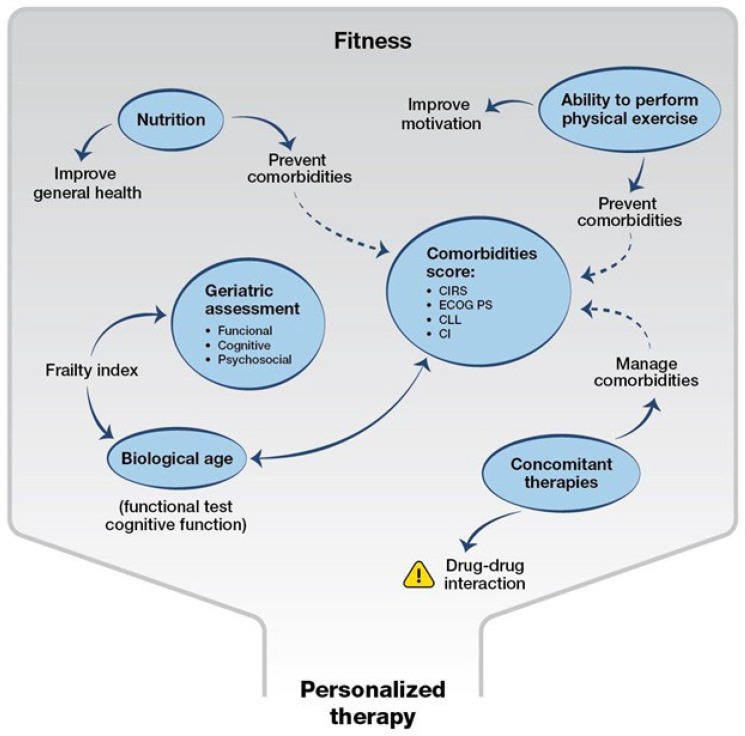
Towards a novel mindset in the management of patients with CLL. The map summarizes the complexity of clinical assessment of patients with CLL in the modern era.

Different strategies that assess clinical nutrition status and risk should be combined to help patients maintain a good lifestyle, prevent some cardiovascular comorbidities, control symptoms of disease, or reduce the development of complications during therapies, such as the risk of acquiring secondary infections [92,93] (Figure 3).

## 9. Conclusions

To ensure favorable clinical conditions and a response to therapies for CLL, it is now necessary to have a broader and multifactorial concept of fitness that considers biological age as well as the presence of comorbidities and frailties. The traditional definition of fitness is no longer relevant in the current management scenario of patients with CLL. Other factors that should be considered are the ability for physical activity and the potential for a healthy diet, as these can reduce or help manage, for instance, the risk of cardiovascular events, which are common in older adults and especially in CLL patients receiving BTK inhibitor treatment. The prospective validation of novel fitness assessment models that consider all these parameters is necessary.

## Figures and Tables

**Table 1:** Common Comorbidity Indexes (CI) used in cancer patients.

Prognostic Score		Organ Assessment	Time- Consuming	Prospective Validation	CLL Setting	Ref
Cumulative Illness Rating Scale	CIRS	14	Yes	Yes	Yes	[5,22,23]
CLL-Comorbidity Index	CLL-CI	3	No	No	Yes	[4,24,25]
Creatinine/Clearance	CrCL	1	No	No	Yes	[33,34]
Comprehensive Geriatric Assessment	CGA	13	Yes	No	Yes	[28,29]
Charlson Comorbidity Index	CCI	18	Yes	No	Yes	[30,31,32]

**Table 2:** Inclusion criteria and patients’ characteristics in clinical trials.

Trial	Inclusion Criteria	Patient Characteristics
RESONATE2 Ibrutinib arm [7]	Over 65 years ECOG PS 0–2	Median Age 73 years (65–89) ECOG PS 0–1 92% CIRS > 6 31% CrCl < 60 44%
ELEVATE-TN Acalabrutinib arm [31]	Over 65 years or Under 65 with CIRS > 6 or Under 65 with CrCl 30–69 mL/min PS 0–2 Significant cardiovascular disease excluded	Median Age 70 years (66–75) ECOG PS 0–1 92.2% CIRS > 6 11.7% CrCl < 70 2.2%
SEQUOIA Zanubrutinib arm [39]	Unfit for FCR: Over 65 years or Under 65 with CIRS > 6 or Under 65 with CrCl 30–69 mL/min	Median Age 70 years (40–86) ECOG PS 0–1 94% CIRS > 6 26.4%
CLL14 Ven-O arm [8]	CIRS > 6 or CrCl < 70 mL/min	Median Age 72 years (43–89) ECOG PS 0–1 87% CIRS > 6 86% CrCl < 70 59.5%
CLL13 Ven-O arm [40]	CIRS ≤ 6 CrCL ≥ 70 mL/min ECOG PS 0–2	Median Age 62 years (31–85) ECOG PS 0 72% CIRS > 6 0% Median CrCl 86.3 (41.5–180.2)
GLOW Ibrutinib–venetoclax [38]	Over 65 years or Under 65 with CIRS > 6 or Under 65 with CrCl < mL/min ECOG PS 0–2	Median Age years 71 (47–91) ECOG PS 0 33% CIRS > 6 69.8% Median CrCl 66.5 (34.0–168.1)
CAPTIVATE-FD Ibrutinib–venetoclax [41]	Under 70 years ECOG PS 0–1	Median Age 60 years (33–71) ECOG PS 0–1 100%
AMPLIFY [42] acalabrutinib–venetoclax–obinutuzumab	ECOG PS 0–2	Median age 61 years

**Table 3:** Drug–drug interactions of BTK inhibitors and venetoclax.

BTK Inhibitors	Ibrutinib	Acalabrutinib	Zanubrutinib
Strong CY3A4 INHIBITORS	Interrupt dose	Interrupt dose	May reduce dose to 80 mg/bid
Moderate CY3A4 INHIBITORS	280 mg of 140 mg/die up to 70 mg/die (high dose)	100 mg/die	80 mg/bid
Mild CY3A4 INHIBITORS	No change	No change	No change
Strong CY3A4 INDUCERS	Avoid concomitant use	Increase dose by 200 mg × 2/die	Avoid concomitant use
Moderate CY3A4 INDUCERS	No indications	No indications	No indications

## Data Availability

Data availability is not applicable to this article as no new data were created or analyzed in this study.

## Abbreviations

The following abbreviations are used in this manuscript:

BTK	Bruton Tyrosine Kinase
CI	Comorbidity Index
CLL	Chronic Lymphocytic Leukemia
DDI	Drug–Drug Interaction
EFS	Event-Free Survival
OS	Overall Survival
PFS	Progression-Free Survival
